# What is your diagnosis?

**DOI:** 10.4274/jtgga.2017.0009

**Published:** 2017-06-01

**Authors:** Vatsla Dadhwal, Kavita Khoiwal, Aparna Sharma, Dipika Deka

**Affiliations:** 1 Department of Obstetrics and Gynecology, All India Institute of Medical Sciences, New Delhi, India

A para1 woman aged 27 years presented with a 5-month history of heaviness and abdominal distension. The distension had gradually increased over time. There was no history of fever or weight loss. Bowel and urinary habits were normal. Her menstrual cycles were regular. She underwent a cesarean delivery 1 year previously. Intraoperative notes showed an 11x9x8-cm fundal fibroid, her ovaries were normal. The cesarean section was uneventful and myomectomy was not attempted at that time. The post-operative period was uneventful.

When she visited our outpatient department, she was carrying ultrasonography (USG) and magnetic resonance imaging (MRI) reports, which were suggestive of a dermoid cyst or mucinous ovarian tumor. Her hemoglobin was 9.3 gm/dL, the total leukocyte count (TLC) was 6000 cells/mm^3^, and markers for ovarian tumor such as Ca-125 were negative. The MRI films are shown in Figure 1.

On general physical examination, the patient was maintaining good health. Her vitals were stable. In her abdominal examination, a healed cesarean Pfannenstiel scar was seen, there was a cystic mass filling the whole abdomen, the lower limit could not be reached, and mobility was restricted. The abdomen was not tender. In the vaginal examination, the uterus was bulky and the same mass was felt through the anterior fornix.

## ANSWER

**Figure 2:**
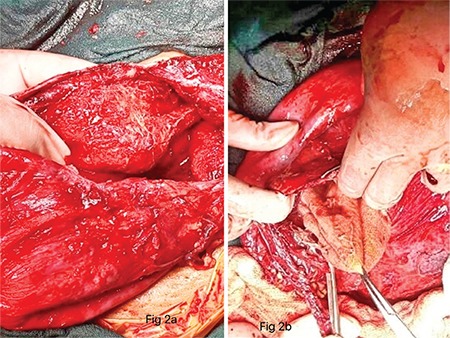
Intraoperative findings (a) A pseudocyst wall that contained 5 liters of pus with retained sponge in situ (b) A retained surgical sponge (held with artery forceps)

The plan was unilateral ovariotomy and staging if the mass appeared malignant because imaging showed tumor confined to one ovary and the patient was P1L1. The possibility of malignancy and the treatment plan of optimal cytoreduction in the event of extra-ovarian disease was explained to the patient and husband prior to surgery. On opening the abdomen, a thick-walled cyst extending up to xiphisternum was encountered. There were flimsy adhesions with the omentum. The content of the cyst (pus) was aspirated with a 21-gauge needle. A stab incision was made and 5 liters of pus was drained. The incision was increased and a whole sponge was found and removed from inside (Figure 2). Further mobilization revealed that bowel formed the posterior wall. Both ovaries were seen separately. There was a subserous fibroid 10x8 cm in size. This was an encysted collection of pus around the sponge, which had been retained in the right paracolic gutter at the time of cesarean section. The abscess wall was removed, the part that involved the bowel was left. A wide bore drain was kept in place for 48 hrs. Myomectomy was not performed because the fibroid was subserous and asymptomatic and there was pus. The pus culture was sterile. The patient received broad spectrum antibiotics (piperacillin + tazobactam and metronidazole) intravenous for 7 days. Postoperative recovery was uneventful and the patient was discharged on day 7 in a stable condition.

Retrospectively, when we took the history again, the patient recalled that she had fever during the post-cesarean period, which settled after a course of antibiotics. Reviewing the MRI images, the strand-like structure was actually the sponge in the abscess. Probably a simpler investigation such as X-ray of the abdomen would have picked up the diagnosis. We did not suspect a retained surgical item because the patient did not initially report a history of fever in the postoperative period.

Retained surgical items (RSI) are a preventable cause of patient morbidity and sometimes mortality. It is included in the 27 “never events” released by the National Quality Forum in the United States of America (1). In a systematic review, authors reported 1.32 RSI events per 10,000 surgical procedures (2). Gawande et al. (3) reported 8 risk factors for RSI, which included emergency operation, unexpected change in operation, more than one surgical team involved, change in nursing staff during procedure, body mass index (BMI), volume of blood loss, female sex, and surgical counts. Out of these risk factors, emergency surgery, unplanned change in the operation, and BMI were found as statistically significant.

Sponges are the most common RSI (68%) (4). Retained surgical sponges are known as gossypiboma, which consists of two words: gossypium (cotton) and boma (place of concealment). A retained sponge may lead to two types of reaction. The first is an acute inflammatory reaction, which may present as infection or formation of an abscess surrounding the retained sponge. The other type of reaction is an aseptic fibrous reaction, which includes formation of adhesion and granuloma surrounding the retained sponge, eventually presenting as a mass (5). Wan et al. (6) performed a systemic literature review of retained surgical sponges from 1963 to 2008, including 254 cases. The most common location was the abdomen (56%), followed by the pelvis (18%). Out of 254 cases, 42% presented with pain/irritation followed by palpable mass (27%), fever (12%), and obstruction (9%); 6% of cases were asymptomatic. The most commonly used diagnostic modality was computed tomography (CT) (61%) and X-ray (35%). USG was used in (34%), and MRI was performed in 20% cases. These cases were at high risk for complications such as capsule formation (51%), adhesions (31%), abscess (24%), and fistula formation (20%) (6). Our patient presented with an abscess, possibly secondary to infection in an exudative reaction due to the sponge. What is unusual, however, is a delayed presentation and no symptoms of fever and pain.

In a series of 8 cases by Chopra et al. (7), five patients presented after abdominal hysterectomy, two patients had a history of lower (uterine) segment cesarean section (LSCS) and another one had a history of ovarian cystectomy. The presentation varied among the patients and included fever, abdominal pain, intestinal fistula, pus discharge from the abdominal wound, and a palpable lump in the abdomen.

Palpable masses in the abdomen/pelvis may be confused with soft tissue tumors based in that location (8). Patients usually present weeks to years after primary surgery and the longest duration reported in the literature was 35 years after Billroth I gastrectomy in a male (9).

Often misdiagnosed preoperatively, a high index of suspicion and imaging studies can help in making the diagnosis. Abdominal X-ray is the first-line investigation and CT is the confirmatory imaging test. MRI has no diagnostic use in such cases because the radiopaque marker is neither magnetic nor paramagnetic, hence not visible on MRI (5).

The usual treatment of an RSI is surgical removal through an open or laparoscopic approach (10).

To emphasize, prevention is always better than cure. To decrease the incidence of RSI, a few new technologies have been developed such as radio-frequency chip identification by barcode scanner (11), computer-assisted counting of sponges using barcodes (12), and a data-matrix–coded sponge counting system in addition to standard manual counting protocols (13). Where these facilities are not available, accurate sponge counts once before surgery, twice after surgery, and better communication between members of the surgical team are mandatory to avoid such unnecessary complications. The diagnosis of RSI should be kept in mind in any postoperative patient who presents with pain, infection or palpable mass.

## Figures and Tables

**Figure 1:**
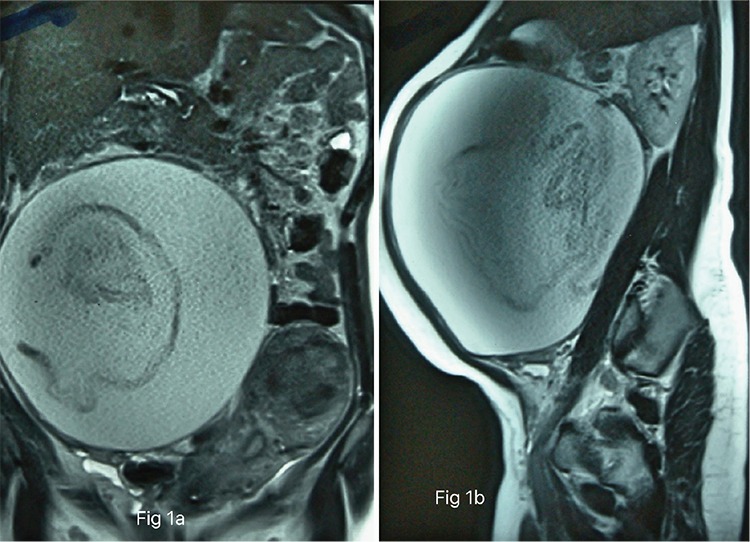
T1-weighted magnetic resonance imaging images (a) Coronal section (b) Sagittal section
